# Pierced Lasso Bundles Are a New Class of Knot-like Motifs

**DOI:** 10.1371/journal.pcbi.1003613

**Published:** 2014-06-19

**Authors:** Ellinor Haglund, Joanna I. Sulkowska, Jeffrey K. Noel, Heiko Lammert, José N. Onuchic, Patricia A. Jennings

**Affiliations:** 1 Center for Theoretical Biological Physics (CTBP) and Department of Physics, University of California at San Diego (UCSD), La Jolla, California, United States of America; 2 Center for Theoretical Biological Physics (CTBP) and Departments of Physics and Astronomy, Chemistry and Biochemistry and Cell Biology, Rice University, Houston, Texas, United States of America; 3 Laboratory of Theory of Biopolymers, University of Warsaw, Warsaw, Poland; 4 Departments of Chemistry and Biochemistry, University of California at San Diego (UCSD), La Jolla, California, United States of America; Wellcome Trust Sanger Institute, United Kingdom

## Abstract

A four-helix bundle is a well-characterized motif often used as a target for designed pharmaceutical therapeutics and nutritional supplements. Recently, we discovered a new structural complexity within this motif created by a disulphide bridge in the long-chain helical bundle cytokine leptin. When oxidized, leptin contains a disulphide bridge creating a covalent-loop through which part of the polypeptide chain is threaded (as seen in knotted proteins). We explored whether other proteins contain a similar intriguing knot-like structure as in leptin and discovered 11 structurally homologous proteins in the PDB. We call this new helical family class the Pierced Lasso Bundle (PLB) and the knot-like threaded structural motif a Pierced Lasso (PL). In the current study, we use structure-based simulation to investigate the threading/folding mechanisms for all the PLBs along with three unthreaded homologs as the covalent loop (or lasso) in leptin is important in folding dynamics and activity. We find that the presence of a small covalent loop leads to a mechanism where structural elements slipknot to thread through the covalent loop. Larger loops use a piercing mechanism where the free terminal plugs through the covalent loop. Remarkably, the position of the loop as well as its size influences the native state dynamics, which can impact receptor binding and biological activity. This previously unrecognized complexity of knot-like proteins within the helical bundle family comprises a completely new class within the knot family, and the hidden complexity we unraveled in the PLBs is expected to be found in other protein structures outside the four-helix bundles. The insights gained here provide critical new elements for future investigation of this emerging class of proteins, where function and the energetic landscape can be controlled by hidden topology, and should be take into account in *ab initio* predictions of newly identified protein targets.

## Author Summary

We discovered a new class of helical bundle proteins with knot-like structures where part of the polypeptide chain is threaded through a covalently bound loop. We call this unique structural motif a Pierced Lasso Bundle. We discovered 12 structurally homologous proteins in the PDB. Our results indicate that the PLB topology is important for regulating the global native state dynamics, especially for a conserved helix that forms part of the canonical receptor interface surface. As a correctly folded structure is necessary condition for function, we explore kinetic folding data to the active conformation and observe two distinct mechanisms to pierce the lasso on route to the native structure, a slipknotting or a plugging event. Threaded elements predominantly slipknot through small covalent loops (50–63 amino acids) while structural components plug through large covalent loops (68–95 amino acids). This information is important for protein design as the loop length and threading mechanism affect dynamics important for function and ease of folding a native structure for potential therapeutic uses.

## Introduction

**Figure 1:**
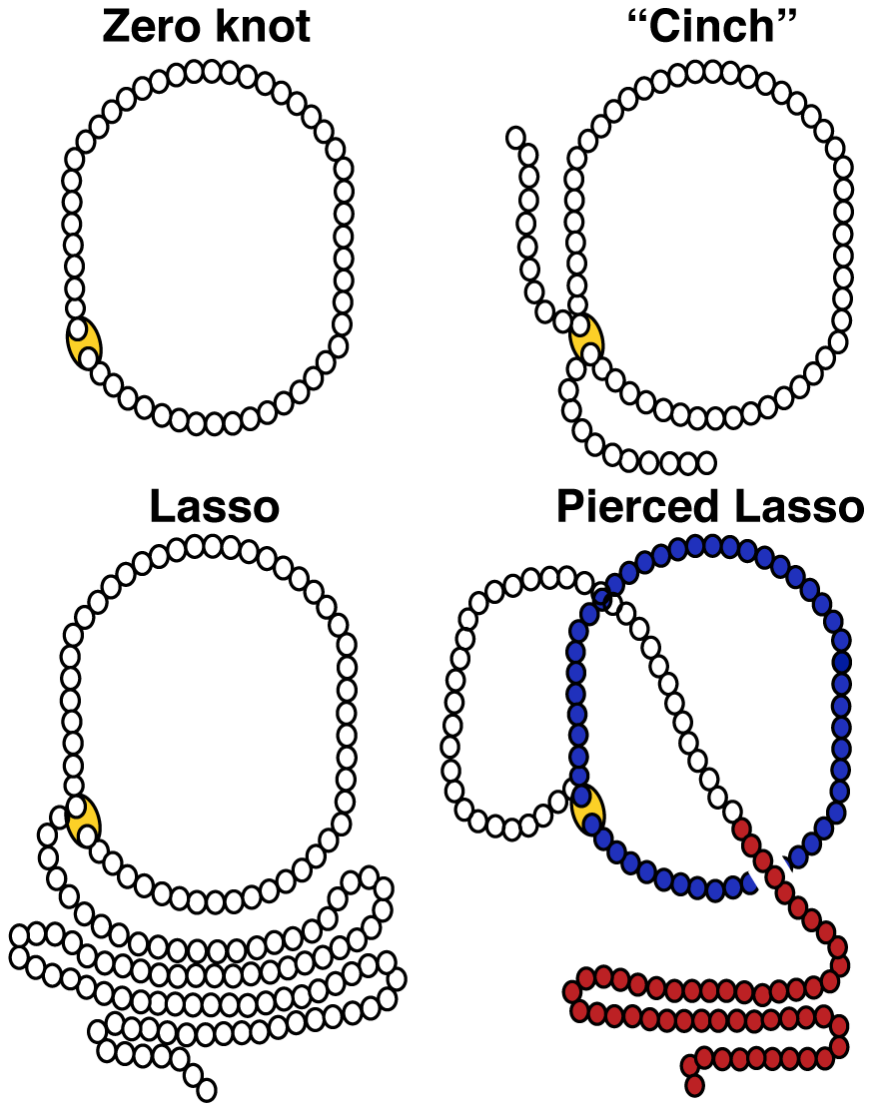
The topology of covalently linked loops in proteins. Closed loops occur in proteins under oxidizing conditions when two cysteines are close enough in space to form a disulphide bridge. The size of the loop depends on the sequence separation between the cysteines forming the closed loop. The simplest knot in mathematics is called a “zero knot” (a closed circle). This occurs in proteins under oxidizing conditions when the N- and C-terminal residues are cysteines. In this case a complete circle will be formed by the polypeptide backbone. When the two cysteines are positioned in the middle of the sequence a so-called “cinch” is formed leaving the two terminals open. When one of the terminal residues is a cysteine a lasso like structure is formed. If there are any parts of the polypeptide chain piercing the covalent loop a pierced lasso is formed. The pierced lasso is colored blue in this figure. The white fragment demonstrates the threaded polypeptide chain and the red part is the terminal in front of the covalent loop.

The four-helix bundle is a common motif in nature [1], [2], [3], [4] often used as a target for designed pharmaceutical and nutritional biomolecules [5], [6], [7]. The cytokine subfamily is a family of four-helix bundles that are soluble proteins secreted from different organs/tissues. Cytokines act as chemical messengers important in intercellular communication. They regulate differentiation, proliferation, activation and death of many cell types, with particular involvement in the regulation of the circulatory system and production of immunity and inflammatory responses [8]. Most four-helix bundles also have conserved cysteines within the motif, whose disulphide bonds help maintain their structure and stability [2]. Every protein containing a disulphide bridge forms a covalently closed loop. When the N- and C-termini are covalently linked you have the simplest knotted topology in mathematics, termed a “zero knot” [9]. The “zero knot” is present in the cytokine Interleukin-36 [10], the θ-defensins as well as other lower organism circular proteins as reviewed in [11] (Figure 1, top left). Nonetheless, a true “zero knot” is rare in the case of proteins. More commonly, the covalent loop creates a “cinch” in the polypeptide chain within the central sequence and the N- and C-terminal ends extend from the internal covalent loop (Figure 1A, top right). Occasionally, either an N- or C-terminal cysteine residue participates in forming the closed loop to generate a “lasso-like” structure (Figure 1, bottom left). The size of the covalent loop depends on the sequence separation between the two cysteines forming the covalent loop. If the size of the loop is big enough, it is possible for part of the polypeptide chain to thread through and create what we term a Pierced Lasso (PL, Figure 1, bottom right).

**Figure 2:**
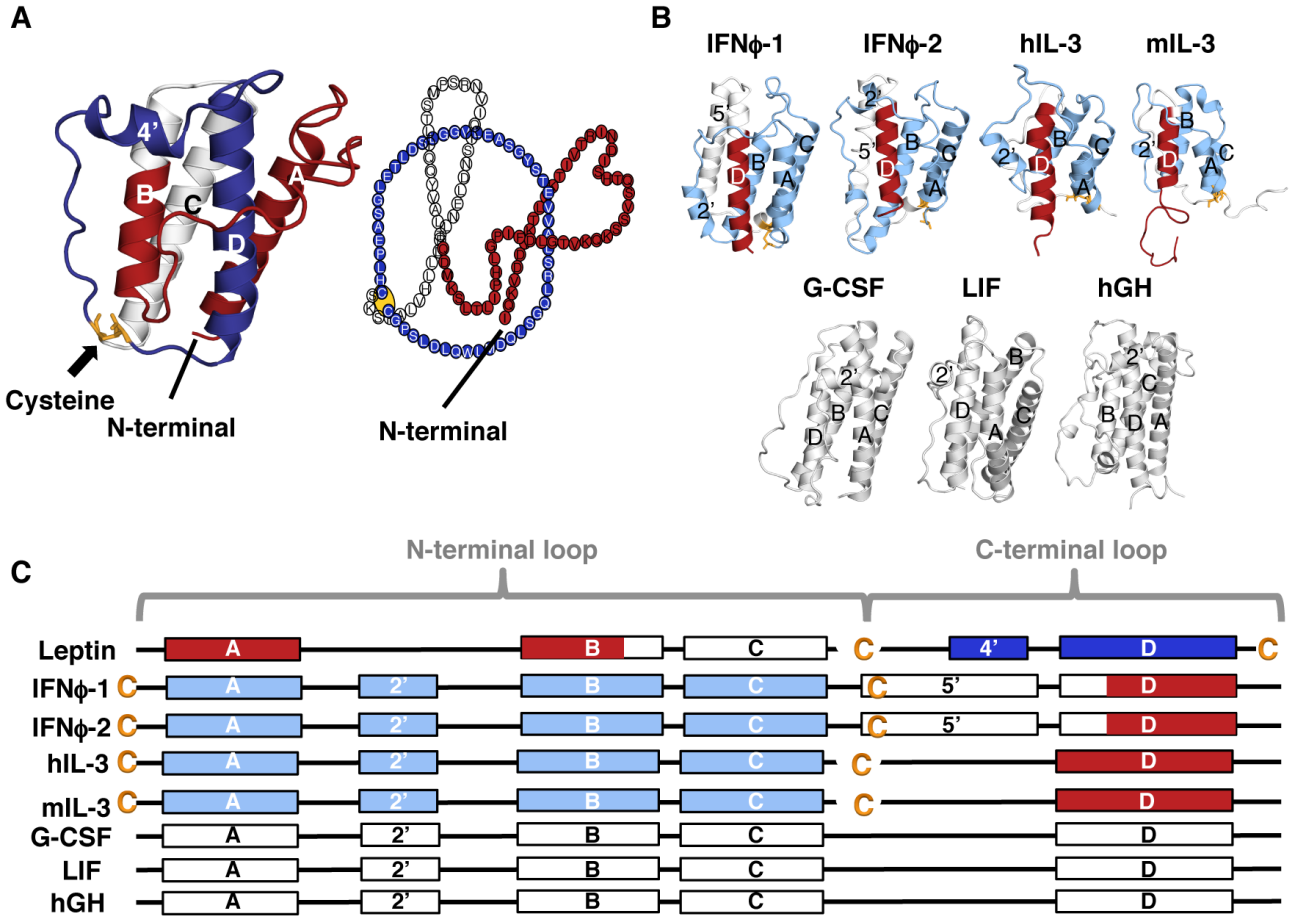
The unique topology of a Pierced Lasso Bundles (PLBs). (A) The native structure of leptin (PDB code 1AX8 [79]). A disulphide bridge is located between residue C96 and the C-terminal cysteine (yellow), creating a covalent C-terminal loop (dark blue). Consequently, a helical hairpin of helix C and half of helix B (white), have to thread through the covalent loop to reach the native state. The N-terminal part stays in front of the covalent loop (red). Hence, the overall conformation of the protein creates a Pierced Lasso Bundle (PLB) topology [12]. (*B*) Four of the new PLBs that we discovered in the PDB plus three unthreaded four-helix bundles are shown in ribbon diagram format. These new PLB proteins have their covalent loop at the opposite end from leptin, creating an N-terminal lasso (light blue). The threaded element (white) is behind the loop from this view while the C-terminal end (red) is in front of the covalent loop. (*C*) A cartoon defining the elements of the different PLBs showing the PL in blue (dark blue for a C-terminal lasso and light blue for an N-terminal lasso). The threaded element, behind the covalent loop, is white and the terminal in front of the loop is red. The unthreaded foud-helix bundles are shown in white.

Recently, we discovered complexity in leptin's fold created by a single disulphide bond [12] between residue C96 and the C-terminal cysteine (C146), which creates a lasso as described in Figure 2A. The folding complexity in leptin comes from threading a helical-hairpin through the closed covalent loop in order to reach the native fold [13], [14], [15], [16] to form a PL Bundle (PLB, Figure 1A). This threading is reminiscent of the more common knotted proteins, where a protein terminal must thread across a twisted loop [15], [16], [17]. In the case of leptin, the structure is analogous to slipknotted proteins, where part of the protein adopts a hairpin-like configuration that threads across the covalent loop (Figure 1A). A slipknotted polypeptide geometry (topology) adds folding complexity that was unrecognized until recently [13], [14], [15], [18], [19], [20], [21]. Since the PL in leptin is distinct from knotted/slipknotted proteins, where the protein backbone ties a knot, and from the cystine knot that is created by at least three disulphide bonds [22], [23], [24], [25], we called this new motif a Pierced Lasso Bundle (PLB, Figure 2A) [12]. This new class of proteins is distinct from previously classified cystine knotted proteins. In the PLB case, a closed covalent loop is created from a single disulphide bridge enclosing one of the terminals with one of the loops where part of the amino acid chain threads through and pierces the covalent loop. In the cystine knotted class, the added complexity beyond a closed loop is created by an additional side-chain mediated chemically cross-linked knot through the covalent loop [28].

PLBs, unlike cystine knots, are able to unfold their threaded elements. Furthermore, unlike knotted/slipknotted proteins, PLBs can modulate their complex topology based on the oxidation conditions of the disulphide bridge. Thus, breaking the bond/contact between the two cysteines also breaks the covalent loop and thereby unthreads the structure. Because of the exciting functional consequences these dynamics may have, we searched for other proteins containing a similar PLB topology. A comprehensive search of the Protein Data Bank (PDB) found 11 structures with a similar threaded motif. Leptin has many structurally homologous proteins where disulphide bridges create a covalent loop, but only 11 had a threaded element through the covalent loop. Interestingly, there is a difference between leptin and the other threaded structures in terms of the location of the closed loop. The covalent loop in leptin is located at the C-terminal end while all other structures, found to date, are knotted at the N-terminal end (Figure 2A and B).

The threaded structure of leptin influences the Native State Dynamics (NSD) and thus the biological activity [12]. Here, we explore the effects of a C- versus N-terminal pierced lasso as well as the folding and the NSD in the related structures. Additionally, we investigate the threading mechanism as the effect(s) of loop size. Structure Based Models (SBMs) were used to study the *human* and *murine interleukin 3* and two *zebra fish interferons* (Figure 2B and C). Additionally, we compare the folding mechanism for three of the unthreaded four-helix bundles (the G-CSF, LIF and hGH, Figure 2B and C), which are members of the leptin family of long-chain helical cytokines. The results show that all PLB proteins stabilize the covalent loop as an initial step in folding (independent of an N- or C-terminal lasso). The disulphide bridge helps stabilize the secondary structure formation that builds the base of the lasso. Remarkably, leptin and mIL-3 mainly slipknot structural components through their lassos, whereas the remaining PLBs thread the C-terminal helix through the N-terminal lasso by a so-called plugging mechanism [26], [27]. We provide, for the first time, direct evidence that the size of the covalent loop influences the threading mechanism. A small loop primarily uses a slipknotting route while the bigger loops are preferentially pierced by a plugging mechanism. In all cases, the N-terminal receptor-binding helix (helix A) is the last element to fold. All PLBs found to date, save leptin, have an N-terminal lasso that pins down the canonical helix A via a covalent linkage, while leptin's helix A has freedom to reorient and fray in the C-terminal PLB. This permutation from the more common N-terminal to C-terminal linkage of the PLBs results in an intriguing switch of the receptor binding helix A from tethered to dynamic and suggests that while the functional landscapes are shared in PLBs, variations in protein-receptor interface dynamics may be needed for signaling activity.

## Results and Discussion

### The cytokine family

**Figure 3:**
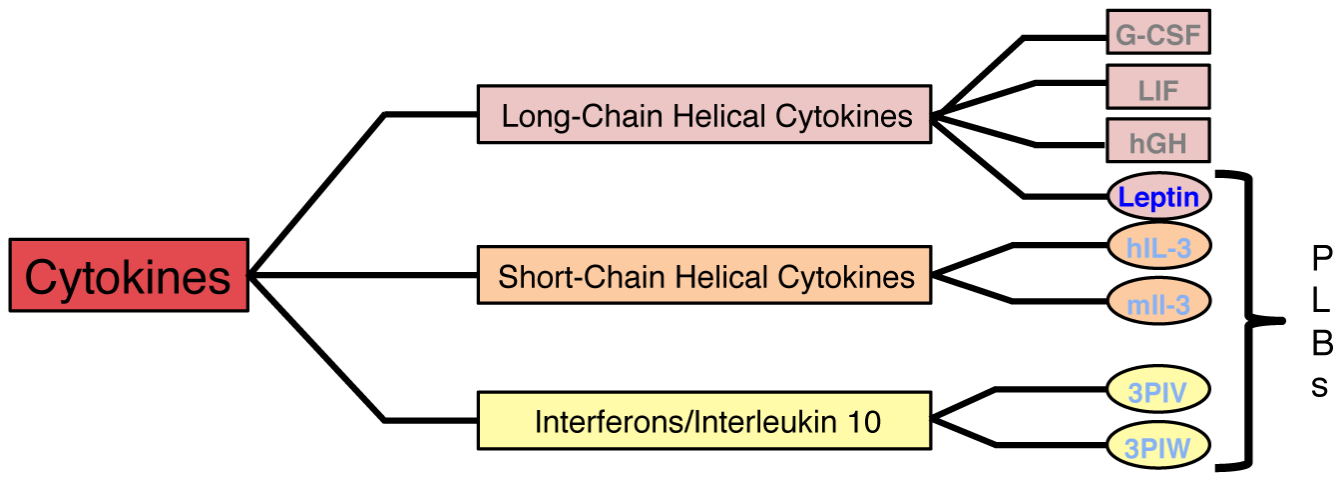
Schematic view of the helical cytokine sub-family. The cytokine sub-family (red) is divided into three families: The long-chain helical cytokines (pink), the short-chain helical cytokines (orange) and *interferons/interleukin* 10 (yellow). The overall topology within the cytokines is conserved with differences in length and topology, where the *interferons* and *interleukin* 10 have two extra helices outside the four-helix bundle and the long-chain and short-chain helical cytokines have one extra helix. The figure shows the proteins discussed in the text, where threaded motifs (PLBs) are circled and unthreaded proteins are boxed. We note that there are many unthreaded examples as well as a few PLB proteins in each family, not shown in this figure.

Cytokines are soluble proteins secreted from different organs/tissues that act as chemical messengers important in intercellular communication. All cytokines bind to a subset of homologous membrane bound receptors, activating similar intercellular signaling pathways [29], [30], [31]. The conserved cytokine motif, a four-helix bundle, indicates that the helical cytokines may have evolved from the same ancestral origin (Supporting Figure S1 and Supporting Table S1). Despite the structural identities, there are little or no sequence similarities within the family due to co-evolution, where each ligand and its specific receptor have diverged in sequence from its ancestors. Therefore, recognition by commonly used sequence homology methods is not possible [32]. Instead, structural methods are used to classify these four-helix bundles as cytokines. Furthermore, all cytokines share a characteristic up-up-down-down fold, forming a two-layer packing of anti-parallel helix pairs were helix A and D packs against helix C and B. The superfamily of helical cytokines is divided into three families: long-chain helical cytokines, short-chain helical cytokines and *interferons/interleukin 10* (Figure 3) [32]. While the overall geometry of the cytokines is conserved, there are differences in structure such as chain length and secondary structural elements (Supporting Table S1).

The PLB protein motif in leptin is a unique fold for proteins in general. A search of the PDB lead to the discovery of an additional 11 proteins with a similar threaded motif. Here, we compare leptin dynamics and threading to four PLBs, two *Zebra fish interferons*, *human* (hIL-3) and *murine* (mIL-3) *interleukin 3* (Supporting Table S1). Additionally, three unthreaded four-helix bundles were investigated as controls, namely *Granulocyte colony-stimulating factor* (G-CSF), *Leukemia inhibitory factor* (LIF) and *human Growth Hormone* (hGH). Figure 2 displays the various structures as well as a cartoon describing the position of cysteines (yellow) creating the two types of lassos, i.e. the N-terminal loop (light blue) and the C-terminal loop (dark blue). The four canonical helices making up the core of each protein are labeled A–D. Additional helices are numbered from the position in sequence; for example, the extra helix in leptin is the fourth helix in sequence and is labeled 4′.

### Structural alignment of the helical cytokines and comparison to leptin

#### The long-chain helical proteins

All four-helix bundles within the long-chain helical cytokines have high structural similarity, from 2.45–3.78 Å rmsd compared to leptin. These proteins have low or no sequence identity/similarity (from 14.7/23.2% to 20.8/27.8%). All structures have an extra small helix in one of the loops outside the four-helix bundle. Leptin has the extra helix in the C–D loop while G-CSF, LIF and hGH have an extra helix in the A–B loop. Additionally, helix D, the longest helix, has a characteristic bend which is also seen in helix A, B and D for G-CSF, LIF and hGH. Leptin shows a small kink in helix D while the other helices are straight. G-CSF has five cysteines creating two disulphide bridges and one free cysteine, LIF has four cysteines creating one disulphide bond and two free cysteines and hGH has four cysteines forming two disulphide bridges. In all three cases the covalent loop created by the disulphides is “empty”, i.e. without any threaded parts through the loop. The size of the “empty” loop varies from 6 residues in G-CSF to 126 residues in LIF. Overall, all the long-chain helical bundles are structurally similar even though leptin is smaller than the other members and has a threaded motif.

#### The Pierced Lasso Bundle (PLB) proteins

The PLBs have a disulphide-mediated lasso where part of the structure is threaded through the covalent loop. This new class of proteins has low or no sequence identity/similarity (from 10.9/22.6% to 15.1/33.3%, Supporting Table S1) but high structural agreement, from 2.76–4.10 Å (Supporting Table S1). We use four of the 11 new helical bundles (IFNϕ-1, IFNϕ-2, hIL-3 and mIL-3) to investigate the folding and threading route. The *interferons* are larger molecules with two extra helices, one short helix in the A–B loop and a long helix in the C–D loop, while the *interleukins* are shorter and have the extra helix in the A–B loop (Figure 2). *Interferons* and *interleukins* are part of the cytokine subfamily but are classified as members of the *interferons/interleukin* 10 family (Figure 3). These proteins are part of the immune system acting as inflammatory response agonists/antagonists upon pathogen invasion or inflammatory response modulation [33]. There are four significant differences between leptin and all other PLBs (Figure 2): (1) The lasso is formed by the N-terminal end in all PLBs save leptin, which uses the C-terminal end. (2) The size of the lasso varies from 50 residues seen in leptin to 122 in one of the LIFs (Supporting Table S1). (3) The threaded element has different lengths, varying from 10 residues in IFNϕ-1 to 45 residues in leptin. (4) Helix D is the threaded element in all PLBs save leptin, where the helix D is part of its covalent loop. The threaded element in leptin is instead a helical hairpin formed by helix C and part of helix B (Figure 2).

### Folding mechanisms

**Figure 4:**
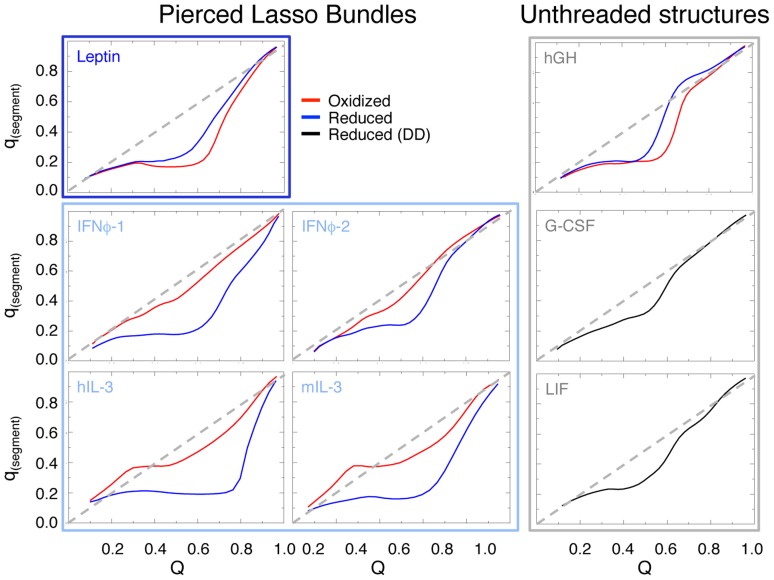
Probability of the formation of helix A. The plot shows data for Q (a measure of nativeness, between 0–1) *versus* q_(segment)_ (formation of a particular secondary structure, in this case helix A). The plots display data for reduced (blue) and oxidized (red) protein in the case of the PLBs and the reduced state (black, DD) for the unthreaded proteins (see method section for a full description). The plots are grouped in three boxes according to position in sequence of the covalent loop, dark blue for the C-terminal covalent loop, light blue for the N-terminal covalent loop and grey for the unthreaded proteins. The plots show that the formation of helix A is a late event on the folding landscape as it starts to build its contacts at high Q. The diagonal dashed grey line shows where q_(segment)_ is tracking the overall folding progress. A significant shift is seen between reduced and oxidized N-terminal PLBs where the oxidized state folds faster than the reduced state.

Structure-based simulations were used to investigate the folding mechanism of the PLB proteins. Two different oxidation states were investigated for the disulphide bridge involved in the lasso, i.e. the reduced state (blue) and the oxidized sate (red) (details in Section Methods). Three unthreaded helical bundles were used as controls and their reduced states (DD, details in Section Methods) are plotted in black. Additionally, both the reduced and oxidized state of hGH were plotted as a control for an unthreaded structure, as it has a large “empty” covalent loop, where nothing is threaded through this element. The folding transition is monitored by the fraction of native contacts formed (*Q*) along the folding trajectory. A native contact is a contact formed between two residues that are close in the native state. *Q* varies from 0, completely denatured, to 1, completely native. The folding mechanism is monitored via *q*_(segment)_, the fraction of native contacts formed by a secondary structure element. *q*_(segment)_ versus *Q* shows the average number of contacts a segment makes as a function of the overall folding progress, and therefore discerns the average order of events during folding. The results are plotted in Figure 4 and Supplementary Figure S2, S3, S4. The diagonal dashed gray line shows where q_(segment)_ is tracking the overall folding progress. The boxes represent the actual positioning of the covalent loop from Figure 2, where light blue represents the N-terminal loop, dark blue the C-terminal loop and gray the unthreaded structures.

#### Comparison of reduced and oxidized PLBs

The PLBs have two different oxidation states of the disulphide bridge controlled by solvent conditions and the distance/space between the cysteines. In the case of PLBs, the structures lose the knot-like topology when the disulphide bond/contact is broken, meaning that the reduced state is actually an unknotted version of the same protein. Interestingly, the order of folding events (mechanism) of leptin is conserved between the two states despite the added complexity in the folding landscape for piercing the C-terminal lasso requirement [12]. The final step in folding of leptin is the formation of helix A (Figure 4). In contrast, the folding of the N-terminal PLBs is altered in the oxidized versus the reduced states (Figure 4 and Supporting Figure S2, S3, S4). For instance, the formation of helix A occurs earlier in the oxidized N-terminal PLBs (Figure 4). This change in kinetics is likely a result of the disulphide bridge pinning helix A to the four-helix bundle in the N-terminal PLBs, adding native contacts in the unfolded state at low values of Q. It is important to note that while the formation of helix A occurs earlier in the oxidized state, it is still the last element to be fully formed in all PLBs. A careful comparison of all the structural elements in the folding of the PLBs indicates that there are also changes in helix C as a function of oxidation state in the N-terminal PLBs (Supporting Figure S3, and S2 and S4 for helix B and D) although they are much smaller than the observed shift in Helix A upon oxidation of the covalent loop. As both helix A and C are part of the closed loop in the N-terminal PLBs, but only part of the threaded element in the C-terminal PLB leptin, the effect of oxidation/reduction is specific to the N-terminal PLBs.

#### Reduced PL bundles versus the unthreaded helical bundles

The PLBs and unthreaded helical bundles are all structurally similar upon reduction. That is, once the additional complexity afforded by the presence of threaded elements is removed by the breaking of the covalent loop, the proteins all display same native topologies (Figure 2). In addition, while the absolute stabilities of the individual proteins vary depending on the size and overall buried surface area, all reduced proteins exhibit decreased stability relative to their oxidized counterparts. The unthreaded and the reduced four-helix bundles also form Helix A last, similar to what is observed for the N- and C-terminal PLBs. Taken together, these results indicate that unlike the β-trefoil cytokines which protect their functional motifs early in folding [34], the helical cytokines fold their receptor engaging Helix A in a late event, likely allowing remodeling during receptor engagement.

#### Piercing the lasso

**Figure 5:**
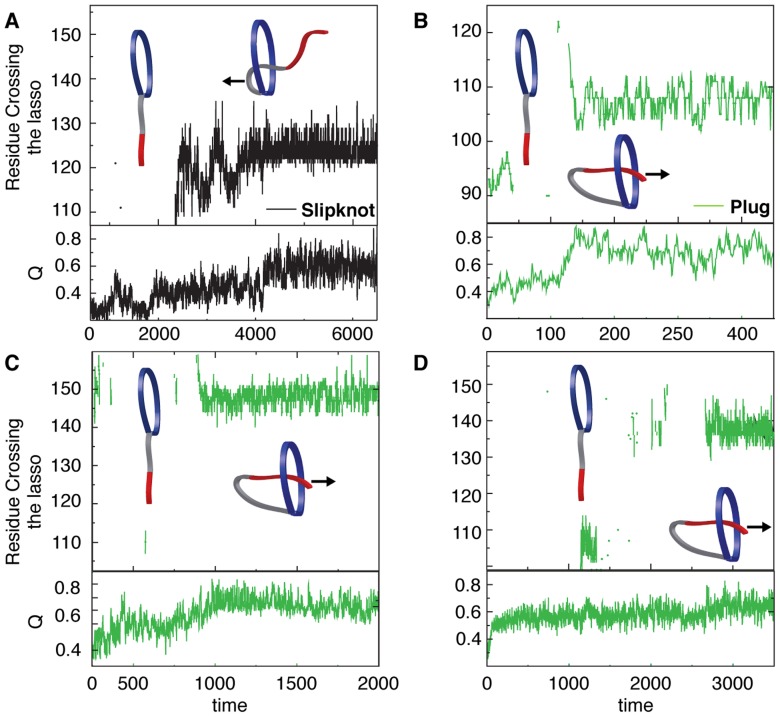
The threading mechanism of the N-terminal PLB proteins. (*A*) The threading mechanism for mIL-3 where slipknotting is the major event (black). We also observe cases where the C-terminal region remains random and no apparent lasso crossing is observed (see cartoon). Panels B-D show the predominance of the plugging mechanism for hIL-3, IFNϕ-1 and IFNϕ-2, respectively. In this mechanism the C-terminal pierces through the N-terminal loop as is shown in the cartoon. As in (A) we also observe cases where the C-terminal region remains random and no apparent lasso crossing is observed (see cartoon).

Threading of a closed loop can occur by two distinct mechanisms: (1) Slipknotting where a hairpin or loop threads through the closed loop or (2) Plugging the free end of the thread through the closed loop. To determine the dominant routes for threading of the oxidized PLBs, we monitored the residue(s) that crosses the covalent loop during folding. Leptin mainly uses a slipknotting mechanism [15], [18] where the helical hairpin of helix C and part of helix B slipknots through the covalent loop [12]. The other possibility of a plugging mechanism is a rare event (less than 1% folding transitions) where the N-terminal plugs through the covalent loop (Supplementary Figure S5). All N-terminal PLBs have a larger covalent loop than leptin (from 63 residues in mIL-3 to 95 in the IFNs, Supporting Table S1). Similar to the dominant slipknotting event in the C-terminal PLB leptin, the N-terminal PLB mIL-3 predominantly uses a slipknotting mechanism (Figure 5A) [35]. However, we observe that the N-terminal PLBs with covalent loops ≥68 residues, predominantly use a plugging mechanism where the C-terminal inserts itself through its covalent loop. Hence, as the loop becomes larger/bigger, the mechanism switches from a slipknot- to a plugging mechanism. This is the first evidence, to date, that directly demonstrates a change in threading mechanism based on covalent loop length in structurally homologous proteins. Smaller covalent loops form more local native contacts with hairpins or turns because these structures allow for building up interaction surfaces that provide the driving force to pierce the lasso via a slipknotting mechanism. Therefore, slipknotting is more likely to occur when threading smaller covalent loops. Increasing the size of the covalent loop increases the “open” space inside the lasso. This leads to a larger number of possible conformations capable of threading a terminal and there are more possible entry encounters with the larger loop that will be productive. As the size of the loop becomes even bigger the terminal is no longer restricted by topological constraints. However in contrast to a polymer [17], [36], which will perform a random motion without forming any native contacts, the protein terminal will make native contacts on the other side of loop and be stabilized, leading to a knot-like native conformation. We use a native-centric model although threading also involves many non-native interactions. It has indeed been shown that including non-native interactions in the model can facilitate knotting [37] and enhance the probability of threading via the entropically unfavorable plugging mechanism [38]. In the case of the smallest knotted protein it was shown by explicit solvent simulation that the energetic roughness along the slipknotting route is not greatly increased by the non-native interactions [39].

### Native State Dynamics (NSD), N-loop versus C-loop

**Figure 6:**
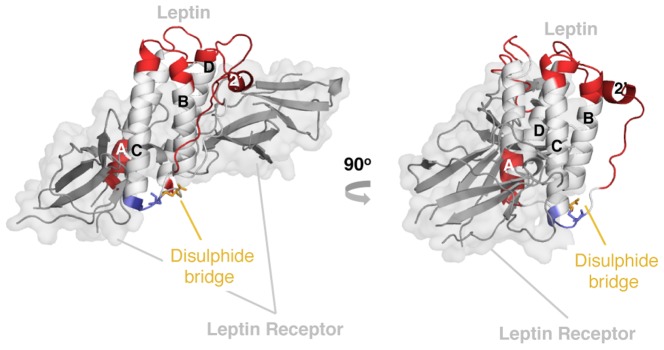
Crystal structure of the leptin receptor complex. The figure shows the leptin receptor complex (PDB code 1AX8 and 3V6O, [40]). The structure of leptin is color-coded according to changes in NSDs, where increased dynamics in the oxidized state is shown in red and increased dynamics in the reduced state is shown in blue. It is clear that there are more dynamics in the oxidized state versus the reduced state. Interestingly, the largest changes in the oxidized state occur at the receptor interface. Previously published results from in vitro activity assays in human cell lines showed that oxidized leptin is more active than reduced leptin [12]. This suggests that leptin benefits from being malleable for receptor interaction.

**Figure 7:**
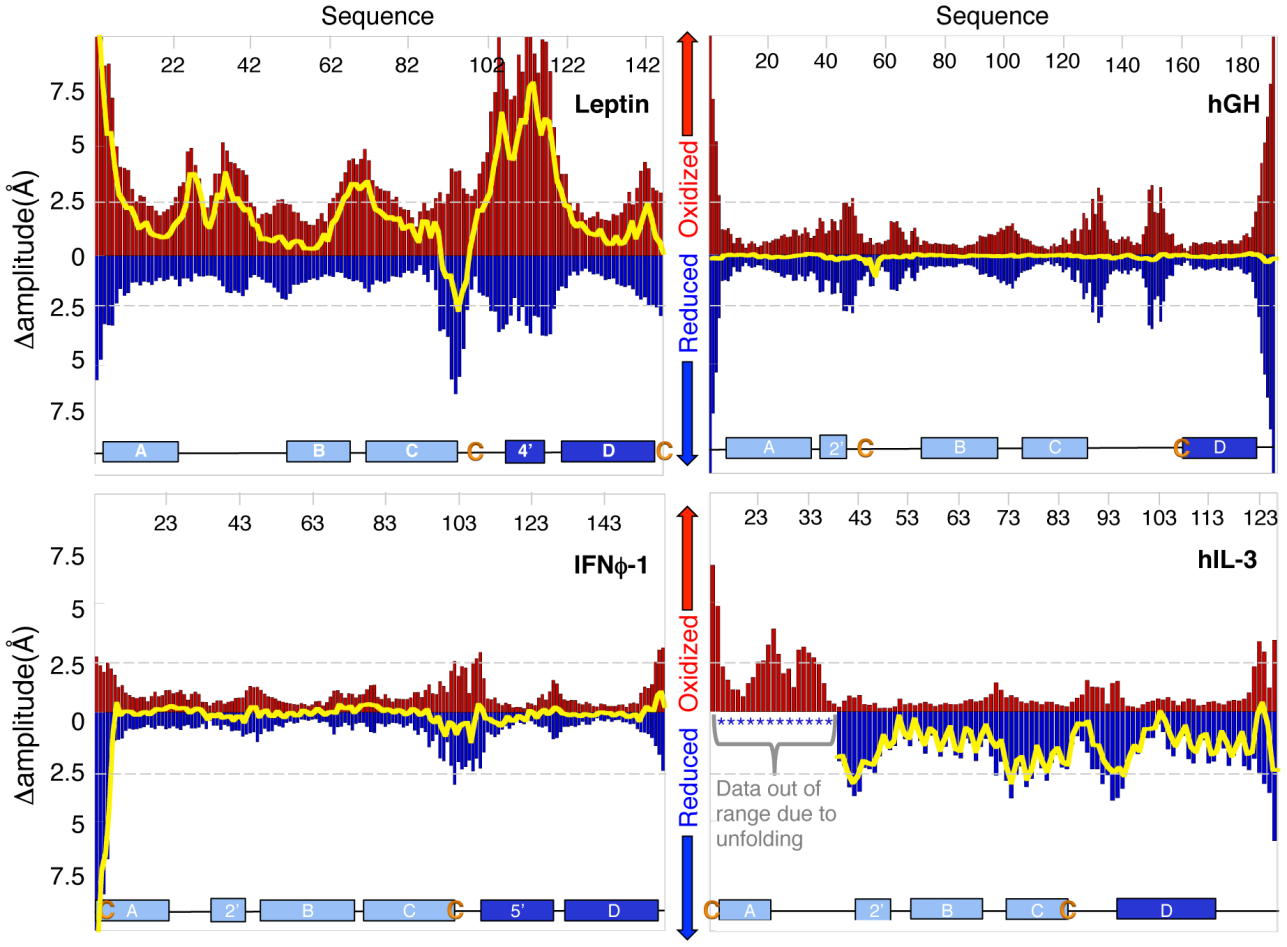
Native state dynamics of leptin, hGH, IFNϕ-1 and hIL-3. Structure based all-atom simulations were performed to obtain NSD. Data for reduced and oxidized protein are shown in blue and red, respectively. The overall fluctuations are shown as bar graphs and the difference between the two states is plotted as a yellow line. The protein sequence is displayed at the top of the graphs and a cartoon of secondary structures is displayed at the bottom (indicating the position of the N- versus the C-terminal loop in light blue and dark blue respectively). Leptin shows an interesting shift in dynamics where the oxidized state is more dynamic than the reduced state over the majority of the structure. The disulphide bridge, not only closes the covalent loop, but also acts as a point of tension inducing dynamics, far from the disulphide bridge. Helix A in leptin shows increased dynamics in the oxidized state, opposite to what is observed in the other PLBs. The decreased dynamics in helix A for the other PLBs is a result of the disulphide bride pining down helix A, thus restricting its dynamics. Interestingly helix A in hIL-3 completely unfolds in the native basin in the reduced state (the data dwarfs the effects on the rest of the sequence and is not included in this figure for clarity). hGH is has an “empty” covalent loop (a “cinch” like structure) of about 100 residues. The plot for hGH shows no significant change of the overall dynamics between reduced and oxidized protein, except for the expected local effect around the disulphide bridge. This implies that that the formation of the covalent loop alone has no effect on the NSD while the presence of a threaded topology piercing the lasso changes the entire protein dynamics.

The NSD for leptin together with *in vitro* activity assays revealed that the disulphide-bond plays an important role in controlling receptor binding [40] and thus biological activity by controlling local motions on distal receptor-binding sites far removed from the disulphide-bridge (Figure 6). These shifts are seen, for example, in helix A as well as in loop four, despite leptin is a C-terminal PLB [12]. To quantify the NSD for the PLBs we performed all-atom structure- based simulations far below the folding temperature, where the protein is effectively always in the folded basin. We calculated the essential dynamics, of the backbone, by projecting the trajectory onto the first four principle components. Oxidation has a significant effect on the amplitude of fluctuation of individual amino acids along the sequence for the PLBs due to topological constraints introduced by the threaded element [41] (Figure 7 and Supporting Figure S5). These modulations in fluctuations are not limited to the regions in the vicinity of the disulphide. Since the disulphide bridge mobilizes helix A, the dynamics of the N-terminal PLBs show the largest shifts. Both *interferons* show additional small increased NSD in the reduced state around the helical hairpin (loop 2, helix B and C), which is part of the closed loop. The *interleukins* show increased dynamics in both terminals in the reduced state. Indeed, in hIL-3 helix A even unfolds completely up on reduction. Taken together, the NSD data indicate that both the *interferons* and *interleukins* are more dynamic in the reduced state. However, leptin is unique in that it introduces ***increased*** dynamics in the ***oxidized*** state. As a control, we performed NSD for the empty covalent loop homolog protein, hGH. This protein shows no significant changes between the oxidized and reduced protein, with the exception of the expected increased local dynamics in the vicinity of the disulphide bridge upon reduction. Taken together, this data indicates that the observed long-range changes in dynamics in the PLBs are a direct result of the effect of a closed loop in the presence of a threaded element of the polypeptide chain. In the case of leptin, increased dynamics distal to the knot in the oxidized state is likely a consequence of the inability to relax the tension introduced into the vicinity of the small closed loop in the lasso. In the other cases discovered to date, local motions are enhanced by increasing the size of the loop and the expected reduction in dynamics upon disulphide bond formation are observed. That is, the simple formation of the closed loop effects only the local but not the global dynamics in the helical bundles, while the presence of a PL alters the global NSDs in both expected and unexpected ways.

### Fraying of helix A and receptor binding

Frustrated surface regions have been proposed as sites relevant for allostery [42]. NSD and frustration in proteins have shown to be essential to protein function [43], [44]. The conserved region for receptor binding within the cytokine family is helix A [45], [46], [47], [48], [49], [50], [51]. The late formation of helix A as well as the fraying of helix A in the reduced state of the PLBs implies that the dynamics is important to receptor binding and activity of the PLBs [12]. As an example, we show the receptor complex of leptin and the changed dynamics between the reduced and oxidized state in Figure 6. This suggests that the malleability of the substrate and receptor interface has been conserved throughout evolution (Supporting Figure S1). More importantly, leptin has kept the malleability in helix A by forming its covalent loop at the C-terminal end. Interestingly, comparing the oxidized and reduced NSD data for leptin reveals a altered dynamics of helix A where the oxidized protein of leptin has greater dynamics then the reduced state. *In vitro* activity assays showed that oxidized leptin is more active than the reduced protein, and supports that a dynamic structure is of importance for biological activity [12]. Nevertheless, all unthreaded helical bundles show reduced dynamic of helix A in general than the PLBs suggesting an overall more rigid structure. Our results suggest that a threaded topology is an important factor designating function. Here we focus on the knot-like four helix bundle class of proteins, however based on the current results we expect that other proteins will have a similar complex topology.

### Future directions

We have found a new class of knotted proteins, namely the Pierced Lasso Bundles (PLBs). The PLB topology is defined as a four-helix bundle where a disulphide bridge closes a covalent loop, and part of the polypeptide chain is slipknotted/plugged through this covalent loop. All PLBs discovered, save leptin, have their covalent loop at the N-terminal end and plug the C-terminal helix through the covalent loop. In contrast, leptin mainly slipknots a helical-hairpin through its C-terminal loop. The closed loop also changes the dynamics of helix A which is important for activity and receptor interaction [45], [46], [47], [48], [49], [50], [51]. The NSD reveals that oxidized leptin is more flexible than the reduced state, implying that dynamics in helix A is biologically important for leptin. Interestingly, sequence alignments of leptin homologs reveal that chicken and turkey leptin has three cysteines, one at the C-terminal end, one at position 100 and one at the N-terminal end [52], [53], [54]. Additionally, natural genetic polymorphism in bovine leptin has developed a sequence with a single cysteine substitution at the N-terminal end (R4C) [52], [53], [54]. This actually allows for three different combinations of covalent loops: (1) Closure of the C-terminal covalent loop, as seen in wild-type leptin, forming a disulphide bridge between residue C96 and C146. (2) The formation of an almost completely circularized protein where residue C4 and C146 form a disulphide bridge, creating a “zero knot”. (3) The formation of an N-terminal covalent loop where residue C4 binds to residue C96. In the latter case, the C-terminal helix could either slipknot or plug through the closed loop. Future *in vitro* experiments can distinguish the three states from each other and the effect of a long N-terminal PLB (90 residues) *versus* the shorter C-terminal PLB as well as investigate fully circularized “zero knot” protein as a template for understanding knot formation and threading control of function in the PLBs (Figure 1). The folding landscape of these three states of leptin could additionally be studied by traditional mutagenic analyses [55], [56], [57]. However, the analysis of the full landscape is complicated by the early threading of the covalent loop that occurs at the level of the transition state. While threading mechanisms have previously been investigated through Fluorescence Resonance Energy Transfer (FRET) [58] this is not an optimal technique in the case of leptin as the loop is 50 residues long and big probes could compromise the threading event. On the other hand, leptin is an optimal system for pulling experiments. For example, a major issue in the field is the inability to untie knots with denaturant [59]. In the case of leptin, simply reducing the disulphide bridge unthreads the structure and we are assured that we are comparing the energetics of the fully threaded and fully unthreaded states. Additionally, reversibly knotting proteins with pulling experiments is extremely complicated [60], while for leptin the experiment is straightforward and can be used to investigate rate of loop threading. Nevertheless, understanding the topological constraints will lead to a broader understanding of the exotic shapes of the free energy landscapes in the growing class of knot-like PLB proteins. Moreover, one should point out that there are probably other undiscovered PL structures deposited in the PDB, where they proteins are bold up of β-strands and/or mixed α/β proteins where the loop is a cinch instead of a lasso. Finally, knowledge about topological constraints in the PLBs could increase the interest of researchers to pharmacologically modulate the pleiotropic hormone leptin [61] and other cytokines, as they have become a hotspot for many medical disorders as cancer, reproduction, diabetes, obesity among others [62], [63], [64], [65]. Taken into account that the PLBs identified here show a different behavior than the unthreaded four-helix bundles suggests that the importance of the threaded element should be considered in modulating receptor ligand interactions for therapeutic development.

## Methods

### Structure Based Models (SBMs)

In this work we used a Cα SBMs [66], [67] to investigate the folding of eight helical cytokines, including five PLB proteins (PDB code 1AX8, 3PIV, 3PIW, 1JLI and 2L3O) and three unthreaded four-helix bundles (1RHG, 1EMR and 1HGU). Each amino acid is represented as a single bead and attractive interactions are given to residue pairs close in the native state. These native interactions are identified based on a shadow map [68], [69]. The basic Hamiltonian is,
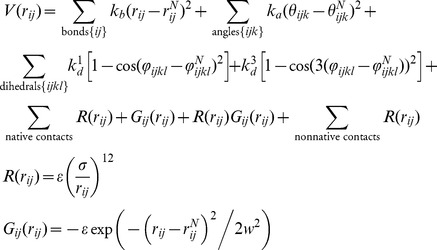



Native interactions have a repulsive term plus an attractive Gaussian term. The R(r*_ij_*)G*_ij_*(r*_ij_*) term is a correction that anchors the minimum of each contact -ε (where the last two corresponds respectively to attractive and repulsive non-bonded interactions [70]. 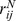 denotes the native distance between atoms *i* and *j* along the sequence. The local topology of the chain is described by the native angles 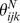 between the bonds connecting residue pairs *ij* and *jk*, and by the native dihedrals 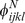 or torsional angles between the planes defined by atoms *ijk* and *jkl*. The strengths of the interactions are given in reduced energy units by the constants k_b_ = 2×10^4^ ε/nm^2^, k_a_ = 40 ε/rad^2^, k^1^_d_ = ε and k^2^_d_ = 0.5ε, where ε is the reduced energy unit. Σ = 4 Å. The details of the model are characterised elsewhere [70], [71].

### Molecular dynamics

We used the web server SMOG (http://smog-server.org/) to create the input files for our simulations [66], [68], [69]. The GROMACS 4.5.3 package was used to perform the molecular dynamics simulations [72]. The integration steps were t = 0.005, stochastic dynamics with coupling constant 2 was used to maintain temperature. The apparent folding temperatures are estimated from each maximum peak in each specific heat curve. For a formed native contact the energy gain is measured by epsilon (*ε*), and thus the temperatures and energies reported in this paper are measured in units of *ε*. For sufficient sampling of the transition states some proteins required umbrella sampling along Q as in [73]. Corrected folding mechanisms (Q versus q_(segment)_) were then created with the Weighted Histogram Analysis Method (WHAM) [74], [75].

### The oxidation state of the disulphide bridge

To mimic the experimental conditions/environment for the disulphide bridge building up the covalent loop we used two comparable *in silico* models, i.e. a reduced state (blue in all plots), an oxidized state (red in all plots). The ability of the disulphide to make and break during folding also was employed to mimic the conditions where folding takes place at the respective reduction potential of the disulphide bridge. This state, the DynamicDisulphide, is best studied in silico where it can be explicitly defined. This state was also simulated for the unthreaded structures (G-CSF and LIF, black in all plots), see Haglund *et al* for a full description of the states of the disulphide bridge [12]. The hGH was simulated as a control for covalent loop formation, as one of the disulphide bridges (C53–C163, forming a so called “cinch”) forms a large “empty” covalent loop of 112 residues. This loop is classified as “empty” as no part of the polypeptide chain is threaded through the loop. This construct can help show the effects of the threaded element in PLBs.

### Native State Dynamics (NSDs)

All-atom structure-based simulations [66], [76] were performed to characterize the NSDs. To investigate the contribution of the threaded topology we performed simulations of both reduced and oxidized states for all PLBs as well as for hGH. A reduced state was simulated for the unthreaded structures (G-CSF and LIF). The slow component of the dynamics described by the first four eigenvector was analysed as described in Haglund *et al* [12].

### Structural and sequence alignment

Some of the crystal structures have gaps in the sequence. Therefore, the Arch pred server [77] was used to recreate the spaces in the structure of leptin, G-CSF and hGH. Due to problems with aggregation the *interleukins* are truncated at the N-terminal end [49], [50] (Supporting Table S1). Also, most of the proteins do not show complete density for the entire sequence as is stated in Supporting Table S1. To align all four-helix bundles with leptin we used the PDB tool “Compare Structures” using the comparison method jFATCAT-ridged and jFATCAT-flexible (http://www.pdb.org/pdb/workbench/workbench.do) [78]. The sequence alignment tools used to align all sequences to leptin were ALIGN Query (http://xylian.igh.cnrs.fr/bin/align-guess.cgi, for sequence identity) and ClustalW multiple sequence alignment (http://www.ebi.ac.uk/Tools/msa/clustalw2/, for sequence similarity). The results from the structural and sequence alignments are shown in Supporting Table S1. To find other proteins with a PLB topology we performed geometrical threading on precompiled all *verses* all input based on the structure of leptin given by jfatcat server. We used a 4 Å rmsd threshold during trace of the fragment matrix. In the second step, we analyzed the discovered structures with P-values lower than 4.0E^−8^ with two conditions: (1) Four-helix motif (all possible combination – motif to thread). (2) Distance along sequence for amino acids which form cysteine bridge has to be bigger than 40 amino acids but shorter than 200. The final set of structures were visually inspected and new motifs were used to repeat the same procedure. Other PL topologies/configurations likely exist; however, they are the subject of future studies as they would reside in a different fold family.

## Supporting Information

Figure S1 **Phylogenetic tree of the cytokines discussed in the text. **The figure shows the average distance tree using percentage identity (PID) on region from ClustalWS alignment of Retrieved from Uniprot. Data analyses indicates that these proteins emerged from a common ancestor (IFN, 2KZ1). The C-terminal PLB leptin is the newest member of the family.
(TIF)

Figure S2 **Probability of the formation of helix B. **The plot shows the same data as Figure 4 where reduced protein is shown in blue and oxidized protein in red. The unthreaded proteins show the reduced state in black (full description in method section). The plots are boxed from the position of the covalent loop with a threaded element, dark blue for the C-terminal loop, light blue for the N-terminal loop and grey for the unthreaded protein (same colours are used as in Figure 4). The formation of helix B is not affected by the threaded topology where all plots shows similar trends.
(TIF)

Figure S3 **Probability of the formation of helix C. **Plotted in the same way as Supporting Figure S2. Helix C seems to be influenced by the formation of the N-loop for the N-terminal PLBs where helix C forms native contacts at lower values of Q.
(TIF)

Figure S4 **Probability of the formation of helix D. **Plotted in the same way as Supporting Figure S2. Helix D shows no significant changes between the oxidized and reduced states.
(TIF)

Figure S5 **The threading mechanism of C-terminal PLB leptin. **(Top) The two possible threading mechanisms are observed in simulations and shown in cartoon format. Slipknotting is the major event (black). We also observe a rare event where the N-terminal plugs through the covalent loop (green). (Bottom) A plot of the progress from unfolded to native (plotted as Q, 0 to 1, respectively) versus time indicates that a slip knotting event progresses more readily to the native state than a plugging mechanism.
(TIF)

Figure S6 **Native state dynamics of IFNϕ-2, mIL-3 and two unknotted four-helix bundles. **Structure based all-atom simulations were performed to obtain NSD. Reduced and oxidized protein is shown in blue and red respectively. The overall fluctuations are shown as bar graphs and the difference between the two states is plotted as a yellow line. The protein sequence is displayed at the top the graphs and a cartoon of secondary structures is displayed at the bottom (indicating the position of the N- versus the C-terminal loop in light blue and dark blue respectively). All PLBs show an excepted shift around the disulphide bridge with additional increased dynamics in the reduced state, when you break the disulphide bridge. The N-terminal PLBs pin down helix A in the oxidized state which decreasing the dynamics significantly in this region.
(TIF)

Table S1 **Structural alignment of leptin compared to PLBs and unthreaded helical cytokines. **We discovered 11 new slipknotted proteins in the cytokine subfamily and compared the structures to leptin: both slipknotted and unthreaded structures were aligned to leptin using the PDB-tool jFATCAT. The results are shown in Supporting Table S1. The cytokines share structural similarities such as the four-helix bundle and the up-up-down-down topology but they have no significant sequence similarity (from 8.7–15.2% sequence similarity compared to leptin).
(DOCX)
