# Supplementary figures and images for: Pierced Lasso Bundles Are a New Class of Knot-like Motifs
Source: PLoS Comput Biol. 2014 Jun 19;10(6):e1003613. doi: 10.1371/journal.pcbi.1003613 (PMC4063663; doi:10.1371/journal.pcbi.1003613)

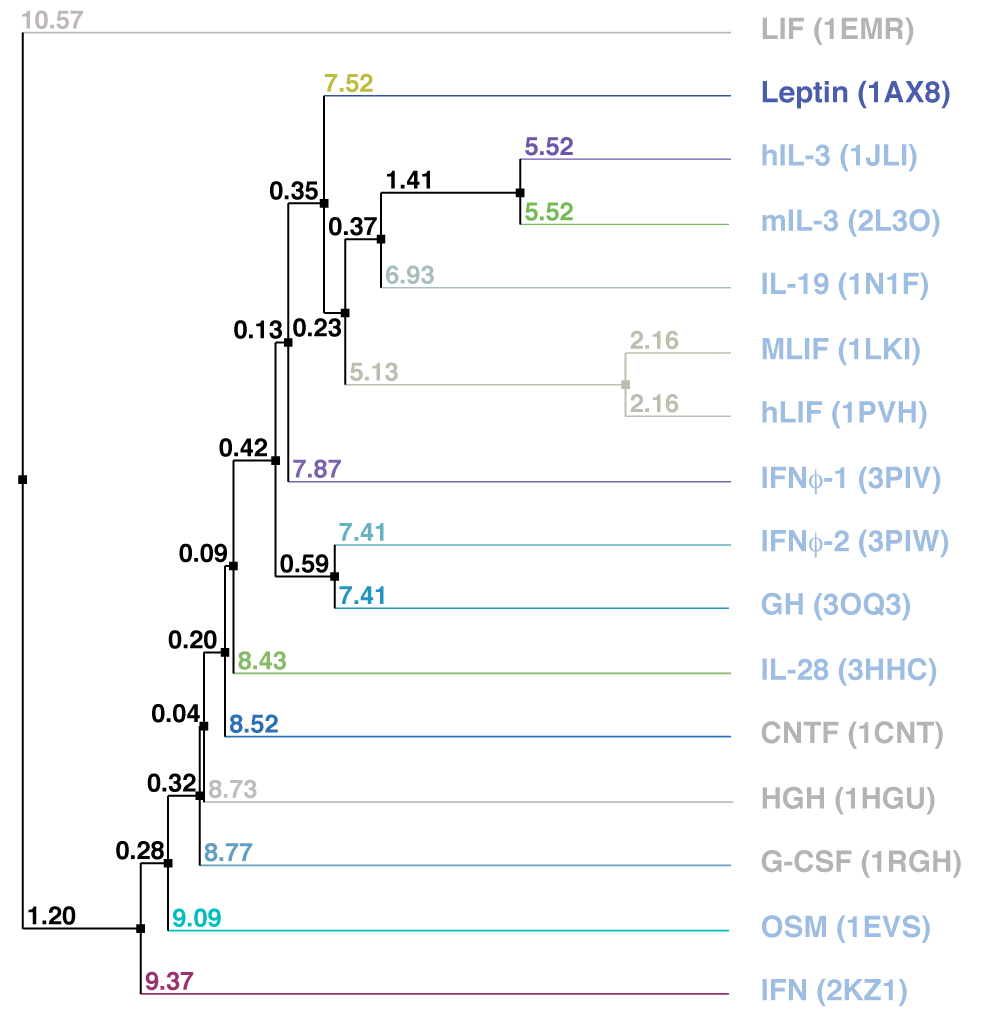

Supplement: Figure S1 — Phylogenetic tree of the cytokines discussed in the text. The figure shows the average distance tree using percentage identity (PID) on region from ClustalWS alignment of Retrieved from Uniprot. Data analyses indicates that these proteins emerged from a common ancestor (IFN, 2KZ1). The C-terminal PLB leptin is the newest member of the family. (TIF) [file pcbi.1003613.s001.tif]

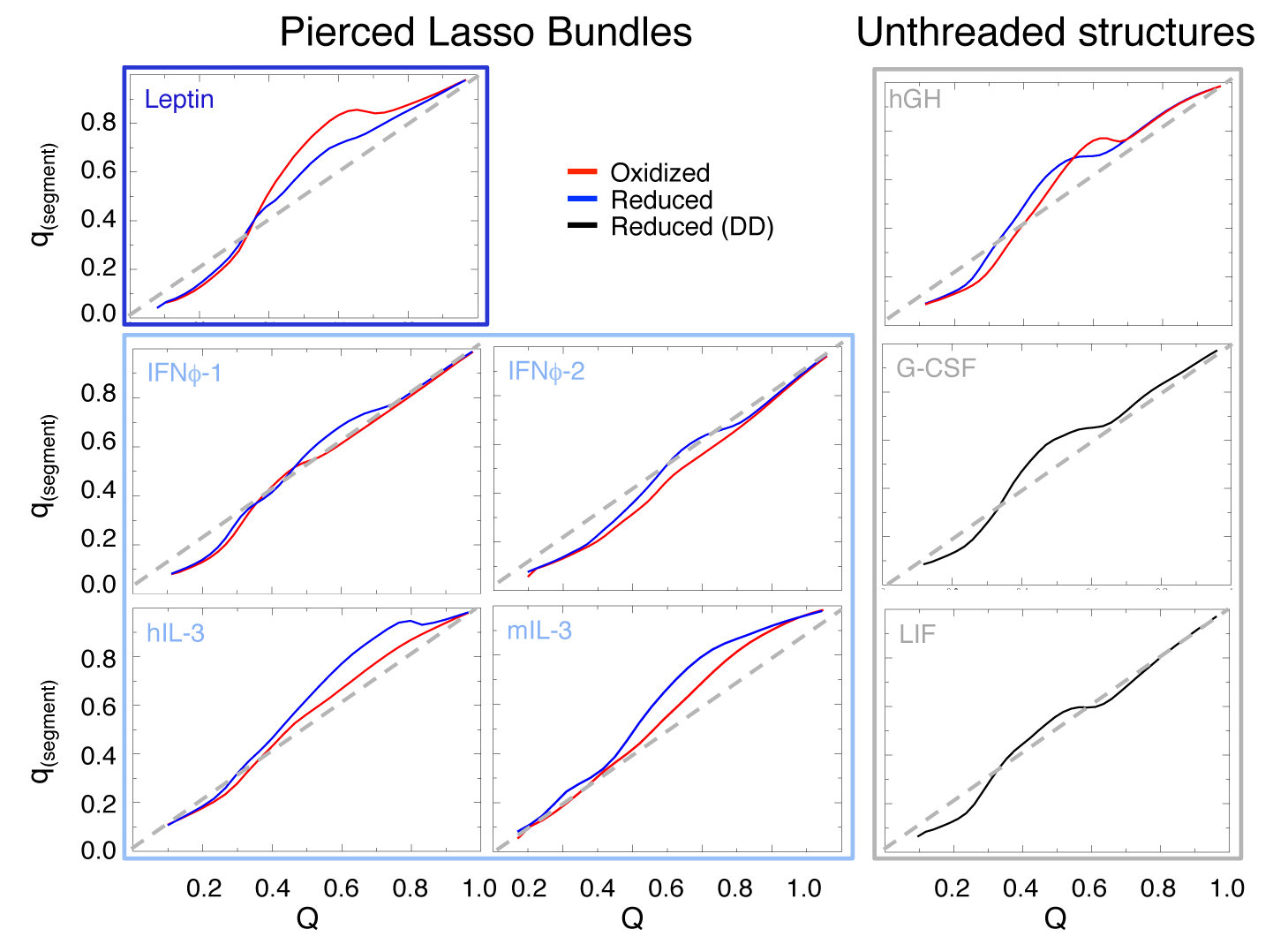

Supplement: Figure S2 — Probability of the formation of helix B. The plot shows the same data as Figure 4 where reduced protein is shown in blue and oxidized protein in red. The unthreaded proteins show the reduced state in black (full description in method section). The plots are boxed from the position of the covalent loop with a threaded element, dark blue for the C-terminal loop, light blue for the N-terminal loop and grey for the unthreaded protein (same colours are used as in Figure 4). The formation of helix B is not affected by the threaded topology where all plots shows similar trends. (TIF) [file pcbi.1003613.s002.tif]

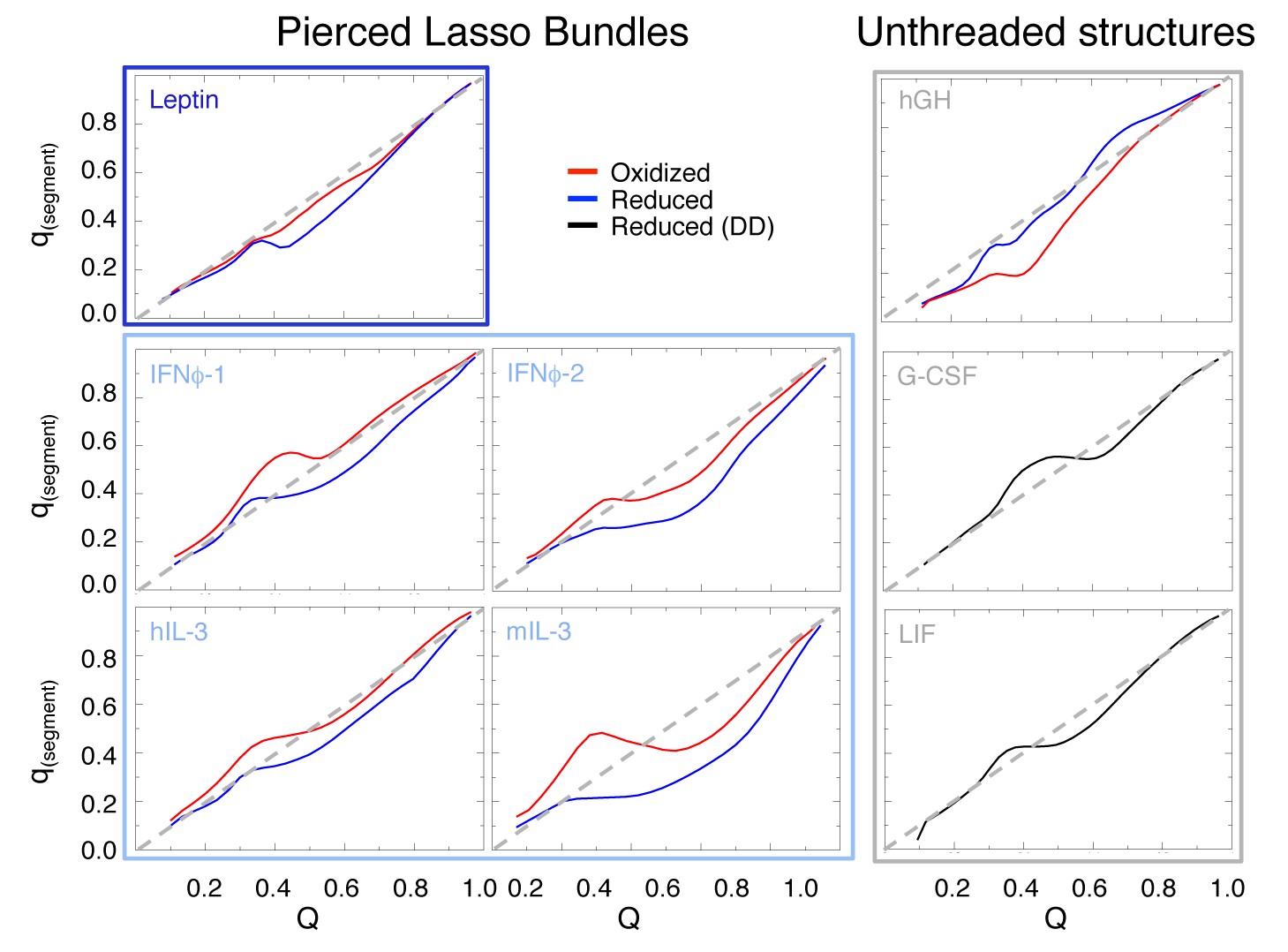

Supplement: Figure S3 — Probability of the formation of helix C. Plotted in the same way as Supporting Figure S2. Helix C seems to be influenced by the formation of the N-loop for the N-terminal PLBs where helix C forms native contacts at lower values of Q. (TIF) [file pcbi.1003613.s003.tif]

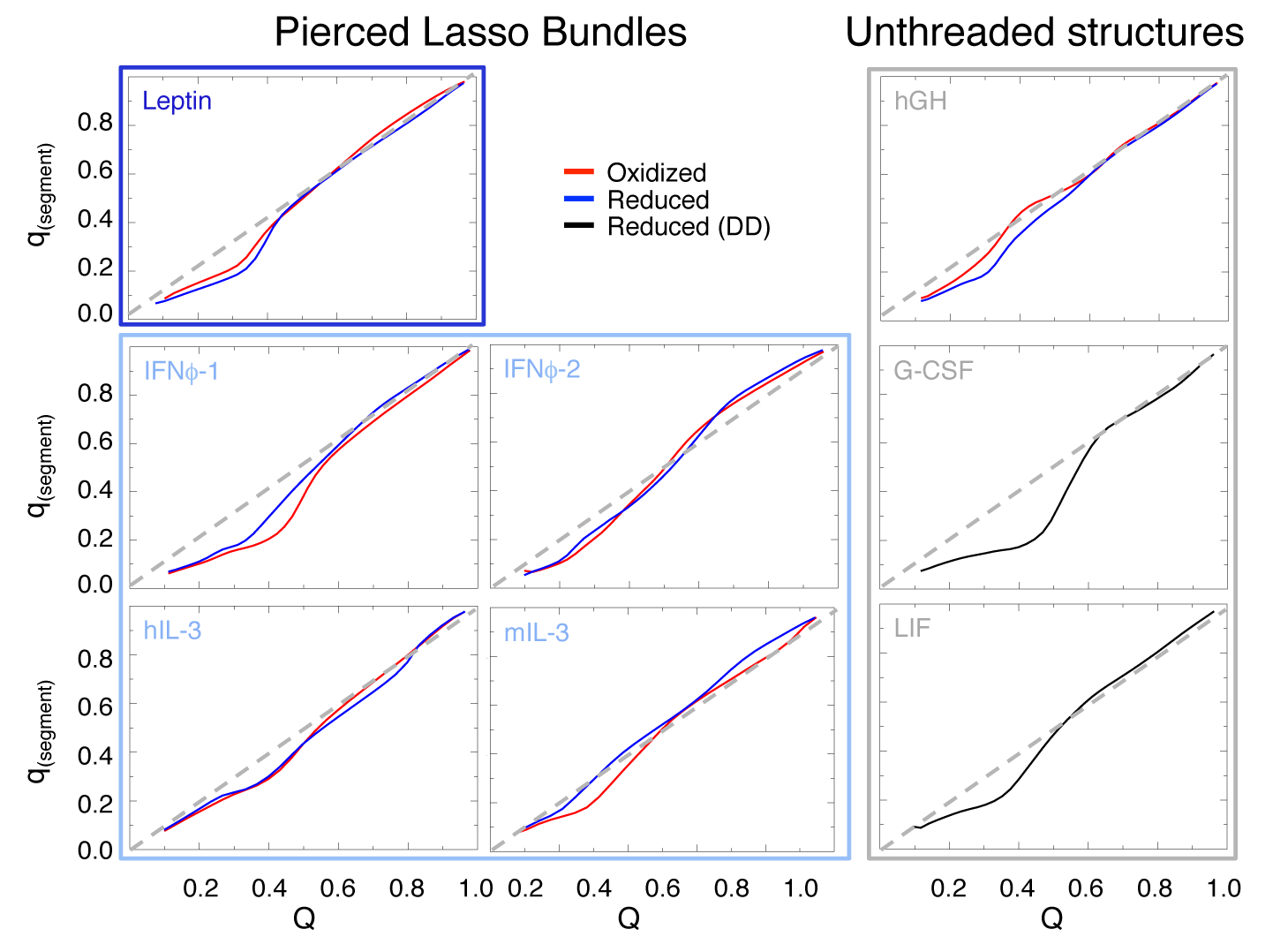

Supplement: Figure S4 — Probability of the formation of helix D. Plotted in the same way as Supporting Figure S2. Helix D shows no significant changes between the oxidized and reduced states. (TIF) [file pcbi.1003613.s004.tif]

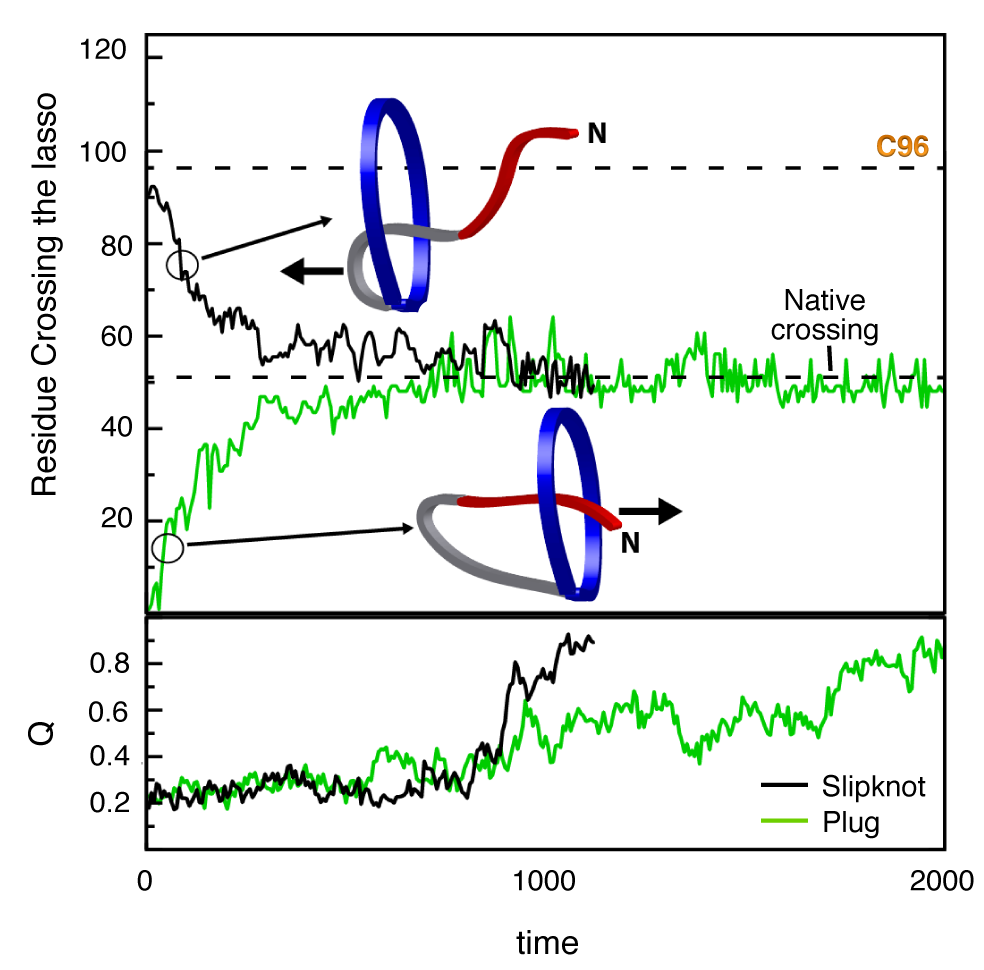

Supplement: Figure S5 — The threading mechanism of C-terminal PLB leptin. (Top) The two possible threading mechanisms are observed in simulations and shown in cartoon format. Slipknotting is the major event (black). We also observe a rare event where the N-terminal plugs through the covalent loop (green). (Bottom) A plot of the progress from unfolded to native (plotted as Q, 0 to 1, respectively) versus time indicates that a slip knotting event progresses more readily to the native state than a plugging mechanism. (TIF) [file pcbi.1003613.s005.tif]

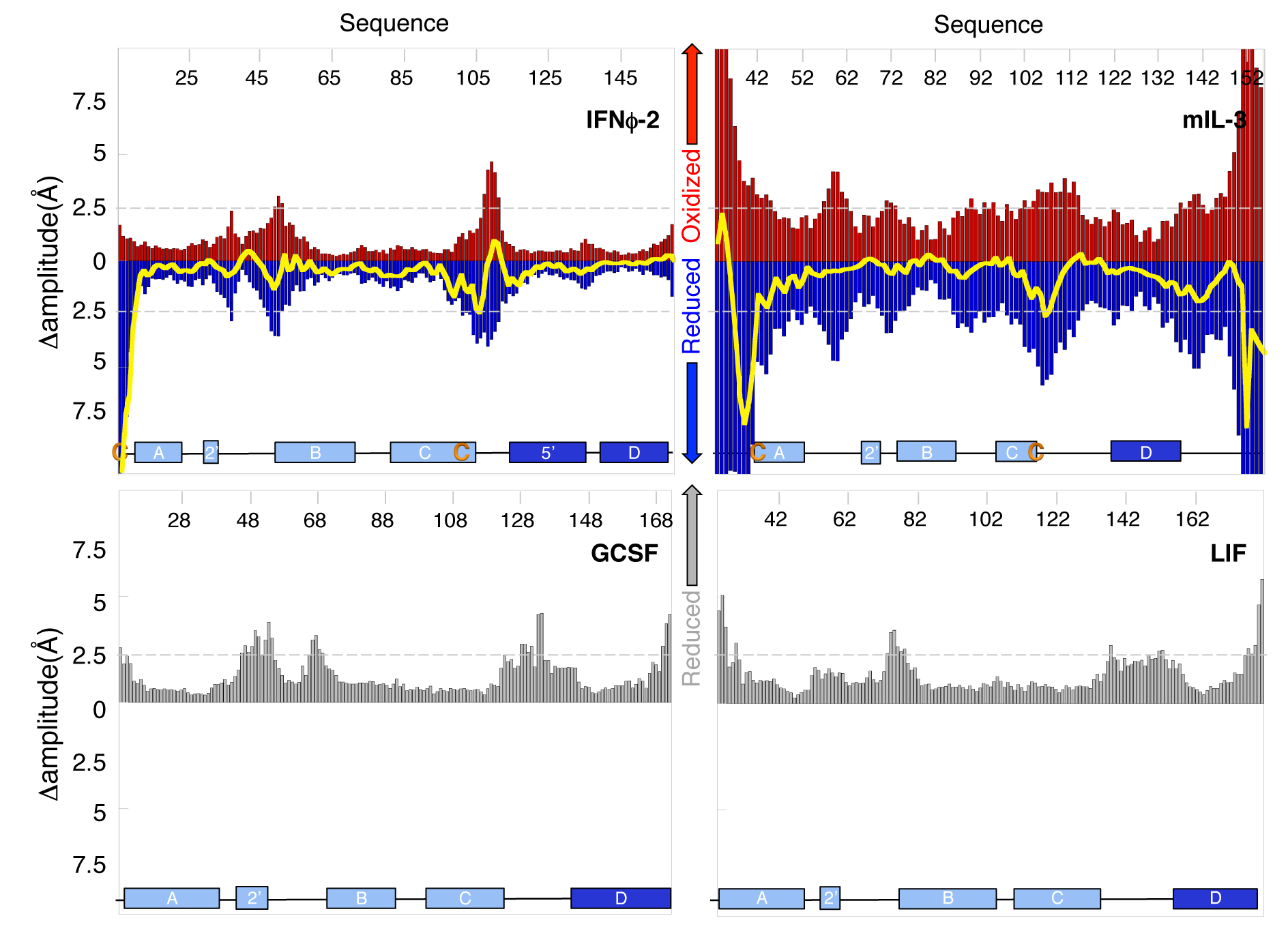

Supplement: Figure S6 — Native state dynamics of IFNϕ-2, mIL-3 and two unknotted four-helix bundles. Structure based all-atom simulations were performed to obtain NSD. Reduced and oxidized protein is shown in blue and red respectively. The overall fluctuations are shown as bar graphs and the difference between the two states is plotted as a yellow line. The protein sequence is displayed at the top the graphs and a cartoon of secondary structures is displayed at the bottom (indicating the position of the N- versus the C-terminal loop in light blue and dark blue respectively). All PLBs show an excepted shift around the disulphide bridge with additional increased dynamics in the reduced state, when you break the disulphide bridge. The N-terminal PLBs pin down helix A in the oxidized state which decreasing the dynamics significantly in this region. (TIF) [file pcbi.1003613.s006.tif]
