# Supplementary material for: Pierced Lasso Bundles Are a New Class of Knot-like Motifs
Source: PLoS Comput Biol. 2014 Jun 19;10(6):e1003613. doi: 10.1371/journal.pcbi.1003613 (PMC4063663; doi:10.1371/journal.pcbi.1003613)
Supplement: Table S1 — Structural alignment of leptin compared to PLBs and unthreaded helical cytokines. We discovered 11 new slipknotted proteins in the cytokine subfamily and compared the structures to leptin: both slipknotted and unthreaded structures were aligned to leptin using the PDB-tool jFATCAT. The results are shown in Supporting Table S1. The cytokines share structural similarities such as the four-helix bundle and the up-up-down-down topology but they have no significant sequence similarity (from 8.7–15.2% sequence similarity compared to leptin). (DOCX) [file pcbi.1003613.s007.docx]

| **PDB code** | **Domain** | **Family** | **Length** | **Alignment Rigid/Flex**  **(RMSD)** | **p-value**  **Rigid or Flex** | **Score** | **Sequence**  **Identity**  **Similarity (%)** | **Loop**  **Size** | **Threaded**  **Element** | **Crystal structure starts at residue** |
| --- | --- | --- | --- | --- | --- | --- | --- | --- | --- | --- |
| [1AX8](http://www.rcsb.org/pdb/explore/explore.do?structureId=1ax8) | Leptin | *Long-chain helical cytokine* | 146 | 3.09  2.83 | 9.4e^-5^  4.3e^-4^ | 160.7  168.4 | - | 50 | 45 | 3 |
| [3PIV](http://www.rcsb.org/pdb/explore/explore.do?structureId=3PIV) | IFNφ-1 | *Interferon/*  *Interleukin 10* | 164 | 3.05  3.05 | 1.6e^-6^  1.6e^-7^ | 206.4  206.4 | 18.8  27.2 | 95 | 10 | 4 |
| [3PIW](http://www.rcsb.org/pdb/explore/explore.do?structureId=3PIW) | IFNφ-2 | *Interferon/*  *Interleukin 10* | 161 | 3.09  2.83 | 9.4e^-5^  4.3e^-4^ | 160.7  168.4 | 15.6  32.0 | 95 | 13 | 6 |
| [1JLI](http://www.rcsb.org/pdb/explore/explore.do?structureId=1JLI) | hIL-3 | *Short-chain helical cytokines* | 112 | 3.53  3.53 | 5.3e^-5^  2.0e^-5^ | 152.7  154.1 | 15.1  26.7 | 68 | 18 | 14 |
| [2L3O](http://www.rcsb.org/pdb/explore/explore.do?structureId=2L3O) | mIL-3 | *Short-chain helical cytokines* | 127 | 3.04  3.04 | 3.6e^-4^  1.4e^-4^ | 133.8  133.8 | 20.8  25.4 | 63 | 28 | 33 |
| [2KZ1](http://www.rcsb.org/pdb/explore/explore.do?structureId=2KZ1) | IFN | *Interferon/*  *Interleukin 10* | 165 | 3.01  3.01 | 1.2e^-5^  1.4e^-6^ | 185.8  185.8 | 20.0  30.7 | 97 | 21 | 1 |
| [3OQ3](http://www.rcsb.org/pdb/explore/explore.do?structureId=3OQ3) | GH | *Long-chain helical cytokine* | 166 | 3.23  3.23 | 1.2e^-5^  1.3e^-6^ | 195.1  195.1 | 17.9  33.3 | 98 | 21 | 1 |
| [1EVS](http://www.rcsb.org/pdb/explore/explore.do?structureId=1EVS) | OSM | *Long-chain helical cytokine* | 187 | 3.20  3.03 | 9.8e^-6^  1.2e^-4^ | 198.4  198.4 | 16.4  28.5 | 121 | 14 | 4 |
| [1LKI](http://www.rcsb.org/pdb/explore/explore.do?structureId=1LKI) | mLIF | *Long-chain helical cytokine* | 180 | 3.98  2.76 | 7.5e^-6^  1.4e^-5^ | 212.0  212.0 | 17.7  28.6 | 122 | 15 | 9 |
| [1PVH](http://www.rcsb.org/pdb/explore/explore.do?structureId=1PVH) | hLIF | *Long-chain helical cytokine* | 201 | 3.11  3.11 | 2.9e^-6^  2.4e^-7^ | 209.8  209.8 | 14.9  26.4 | 122 | 17 | 12 |
| [1N1F](http://www.rcsb.org/pdb/explore/explore.do?structureId=1N1F) | IL-19 | *Interferon/*  *Interleukin 10* | 159 | 4.10  3.66 | 3.8e^-5^  5.4e^-6^ | 184.5  184.5 | 14.7  22.6 | 93 | 20 | 4 |
| [3HHC](http://www.rcsb.org/pdb/explore/explore.do?structureId=3HHC) | IL-28 | *Interferon/*  *Interleukin 10* | 196 | 3.57  3.23 | 1.1e^-4^  2.3e^-4^ | 170.1  182.5 | 17.9  27.0 | 99 | 25 | 1 |
| [1RHG](http://www.rcsb.org/pdb/explore/explore.do?structureId=1RHG) | G-CSF | *Long-chain helical cytokine* | 174 | 3.41  3.41 | 3.3e^-7^  3.6e^-8^ | 223.6  223.6 | 17.4  26.7 | - | - | 9 |
| [1EMR](http://www.rcsb.org/pdb/explore/explore.do?structureId=1EMR) | LIF | *Long-chain helical cytokine* | 159 | 3.78  3.78 | 3.5e^-6^  3.8e^-7^ | 207.5  207.5 | 17.1  24.6 | - | - | 23 |
| [1HGU](http://www.rcsb.org/pdb/explore/explore.do?structureId=1HGU) | hGH | *Long-chain helical cytokine* | 191 | 3.05  2.45 | 1.8e^-4^  2.8e^-3^ | 159.5  159.5 | 19.1  27.8 | - | - | 2 |
| [1CNT](http://www.rcsb.org/pdb/explore/explore.do?structureId=1CNT) | CNTF | *Long-chain helical cytokine* | 187 | 3.03  3.03 | 4.6e^-8^  3.1e^-9^ | 242.4  242.4 | 17.0  23.1 | - | - | 11 |

IL: *Interleukin*

OSM: *Oncostatin M*

IFN: *Interferon*
LIF: *Leukemia Inhibitory Factor*

SCF: *Stem Cell Factor*

G-CSF: *Granulocyte C S Factor*

hGH: *human Growth Hormone*

CNTF: *Ciliary Neurotrophic Factor_­_*
